# Antiepileptic drugs for the treatment of neuropathic pain: A systematic review

**DOI:** 10.4317/medoral.18001

**Published:** 2012-05-01

**Authors:** Maríam L. Vargas-Espinosa, Gemma Sanmartí-García, Eduardo Vázquez-Delgado, Cosme Gay-Escoda

**Affiliations:** 1 DDS. Resident at the Master’s degree Program of Oral Surgery and Implantology. Faculty of Dentistry of the University of Barcelona, Spain; 2 DDS. Master’s degree in Oral Surgery and Implantology, Faculty of Dentistry of the University of Barcelona, Spain. Professor at the Oral Surgery and Implantology Department, and at the Faculty of Dentistry of the University of Barcelona, Spain. Researcher of the IDIBELL Institute. University of Barcelona, Spain; 3 DDS. Chief Professor at the Master’s degree Program of TMJ and Orofacial Pain Unit of the Oral Surgery and Implantology. Faculty of Dentistry of the University of Barcelona, Spain. Orofacial Pain Specialist of the TMJ and Orofacial Pain Unit of the Teknon Medical Center, Barcelona, Spain; 4 MD, DDS, PhD, and Chairman of the Oral Surgery and Implantology Department; Director of the Master’s degree Program of Oral Surgery and Implantology, and of the Faculty of Dentistry of the University of Barcelona. Coordinator-Researcher of the IDIBELL Institute. Chairman of the Oral, Maxillofacial, and Implantology Surgery Department and Co-Director of the TMJ and Orofacial Pain Unit of the Teknon Medical Center, Barcelona, Spain

## Abstract

Many therapies have been proposed for the management of neuropathic pain, and they include the use of different antiepileptic drugs. However, the lack of high quality studies indicates that results on the different neuropathic disorders under study do not recommend a particular drug treatment. This study makes a systematic review of the published literature on the use of several antiepileptic drugs to treat neuropathic pain, and has the objective of considering both its clinical characteristics and pharmacological use, which will depend on their level of scientific evidence and will follow the principles of evidence-based dentistry. The articles were stratified according to their scientific evidence using the SORT criteria (Strength of Recommendation Taxonomy), and it included those articles that only have level 1 or 2. Randomized clinical trials were stratified according to their level of quality using the JADAD scale, an instrument described by Jadad et al. (7). to assess the quality of clinical trials, while studies with a level below 3 were discarded. Recently, type A or B recommendations are given in favor or against the use of antiepileptic drugs to treat neuropathic pain on the basis of their scientific quality.

** Key words:**Neuropathic pain, antiepileptic drugs (AEDs), trigeminal neuralgia, glossopharyngeal neuralgia, post- herpetic neuralgia, burning mouth syndrome, persistent idiopathic facial pain.

## Introduction

Neuropathic pain (NP) has been described by the International Association for the Study of Pain as a pain that is triggered or caused by a primary lesion in the nervous system. A common clinical feature is the lack of a source of nociception. The pain originated inside the nervous structure, and the somatic structures are normal. NPs are usually associated with different neurological symptoms such as burning, hyperalgesia, dysesthesia, or sometimes anesthesia. NP may appear in the form of an episodic or continuous pain (1).

NP can be caused by infections, trauma, metabolic abnormalities, chemotherapy, surgery, radiations, neurotoxins, nerve compression, inflammation and tumor invasion. As to episodic NP, there are different types of neuralgias, such as trigeminal neuralgia , glossopharyngeal neuralgia, geniculate neuralgia and neurovascular pain. And as to continuous NP, the following conditions can be found: peripherally mediated pain (entrapment, deafferentation and neuritic pain), central mediated pain (burning mouth syndrome, atypical odontalgia, post-herpetic neuralgia) and metabolic neuropathies (1).

NP is, regardless of its being central or peripheral, characterized by a neuronal hyper-excitability in the injured areas of the nervous system that may have many characteristics in common with some cellular changes that occur in certain forms of epilepsy (2-5).

Carbamazepine and phenytoin were the first anticonvulsants used in clinical controlled trials. Some studies have shown that these drugs relieve paroxysms in trigeminal neuralgia. Other studies have reported that gabapentin and pregabalin are effective in diabetic and mixed neuropathies, and in post-herpetic neuralgia (2-5).

Lamotrigine, another anticonvulsant drug, has proved effective in trigeminal neuralgia and in post-stroke pain. Many of these drugs have been shown to reduce ectopic discharges in the affected nerve endings and neurons in the dorsal root ganglia by blocking sodium channels (2).

The most common side effects of anticonvulsants are drowsiness and cerebellar symptoms (nystagmus, dizziness). Other side effects observed with carbamazepine and phenytoin are hematological alterations and arrhythmia (2).

There are many drugs that can be used for the treatment of NP, such as carbamazepine, oxcarbazepine, pregabalin, topiramate, gabapentin, clonazepam, lamotrigine, phenytoin, tiagabine and valproic acid. In addition to their antiepileptic properties, these drugs has been used in the treatment of painful and chronic condition, NP included.

Therefore, the aim of this articles is, on account of the large amount of published literature on the treatment of NP, to make a systematic review on the use of different anticonvulsants for the treatment of NP, and on the basis of their level of scientific evidence and the observance of the principles of evidence-based dentistry.

## Material and Methods

A search on articles published by both MEDLINE and COCHRANE was carried out between the years 1966 to 2010. The MeSH (Medical Subject Heading) keywords and headings used were the following entries: “trigeminal neuralgia”, “glossopharyngeal neuralgia”, “post-herpetic neuralgia”, “persistent idiopathic facial pain” (which included “atypical odontalgia”, “phantom tooth pain” and “atypical facial pain”) and “burning mouth syndrome”.

A similar search was made for each of the following terms: “carbamazepine”, “oxcarbazepine”, “pregabalin”, “topiramate”, “gabapentin”, “etiracetam”, “clonazepam”, “lamotrigine”, “phenytoin”, “tiagabine”, “vigabatrin”, “valproic acid” and “phenobarbital”. The literature data identified was then exclusively used in studies in humans, articles written in english, randomized clinical trials and systematic reviews. Both search strategies were in turn combined using the Boolean operator “AND”, as a way to link articles on different NPs and anticonvulsants drugs. The same process was used in the COCHRANE database of the Cochrane Oral Health Group. Three authors analyzed the articles to verify if those obtained in the search were pertinent to the issue under study. Irrelevant articles were discarded. Next, the authors independently stratified the scientific articles according to their level of scientific evidence ( Table 1), using the SORT criteria (Strength of Recommendation Taxonomy), and included those articles that only have SORT level 1 or 2 (6); they subsequently stratified randomized clinical trials (RCT) according to their quality level by using JADAD (Alejandro R. Jadad) criteria (7), and discarded articles with a level below 3. Later, the authors compared their results, which were discussed in the event of disagreement. If no consensus was possible with regard to the level of scientific evidence of any article, a fourth author was included in the discussion. Subsequently, and depending of the scientific evidence, a recommendation was given ( Table 2) either in favor or against the use of this medication for NP.

**Table 1:**
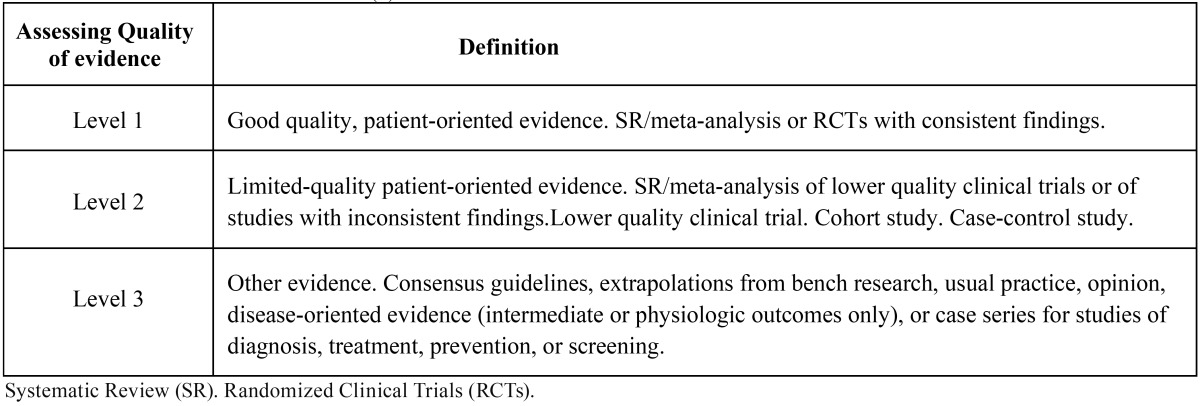
Level of evidence from studies (6).

**Table 2:**
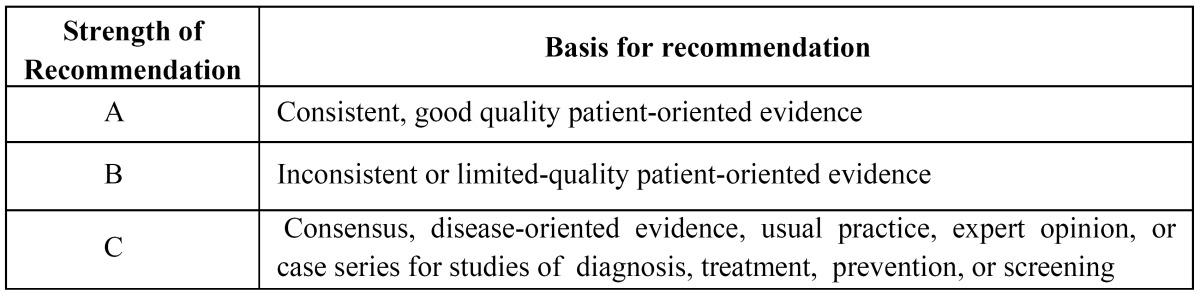
Strength of recommendation grades (6).

## Results

The search on both MEDLINE, using MeSH terms, and the COCHRANE database provided, at a general level, a bank of 84 articles. Sixty articles were discarded for not meeting the inclusion criteria.

Trigeminal Neuralgia (TN)

The search for “Trigeminal neuralgia” and each of the following drugs in the MEDLINE and COCHRANE databases provided the following 55 articles: “carbamazepine”, 27 articles; “oxcarbazepine”, 4 articles; “pregabalin”, 3 articles; “gabapentin”, 3 articles; “topiramate”, 2 articles; “etiracetam”, 1 article; “clonazepam”, 3 articles; “lamotrigine”, 4 articles; “phenytoin”, 7 arti-cles; and “valproic acid” 2 articles. Next, each article was analyzed to determine if they were pertinent to the issue under study.

The 55 articles assessing the effects of different drugs in the treatment of TN were analyzed according to both JADAD and SORT criteria, and only those articles with SORT evidence level 1 or 2, and RCTs with JADAD level above 3 were included.

This analysis produced 10 articles, of which two were systematic reviews and 8 were RTCs that dealt with the effect of carbamazepine in the treatment of TN ( Table 3).

**Table 3:**
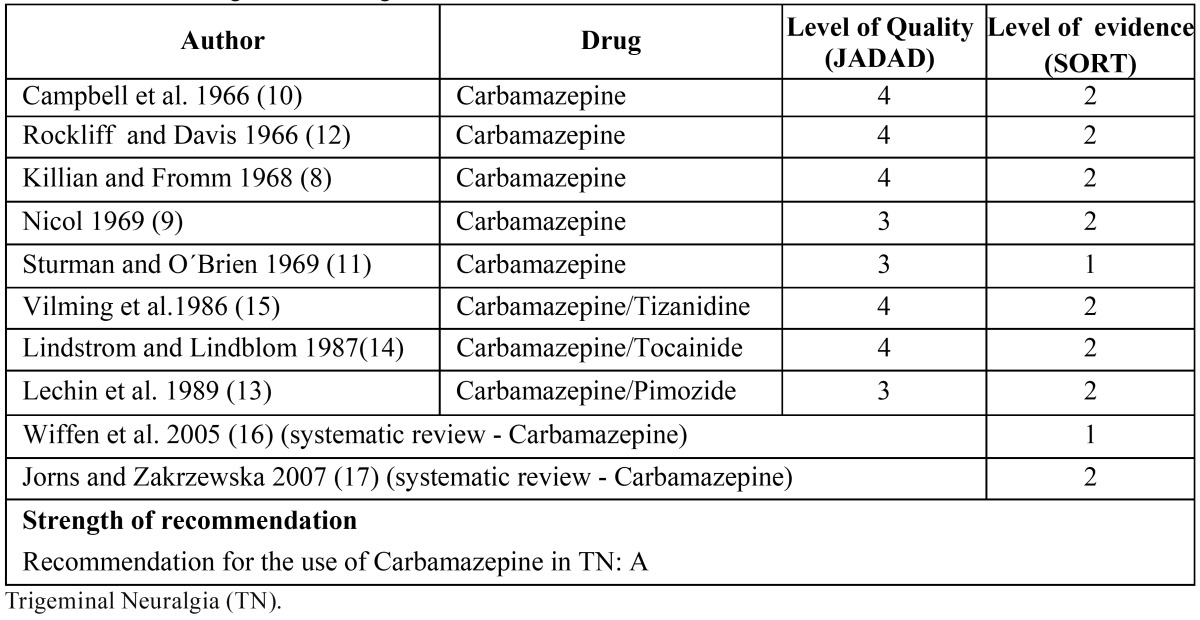
Studies on trigeminal neuralgia.

In five placebo controlled studies by Killian and Fromm (8-12), the authors observed that 19 of 27 of patients with TN had a good response to the use of carbamazepine at a maximum dose of 1 g per day during a 5-day period when compared with placebo. Nicol (9) observed a good response using the same drug at a maximum dose of 2 g per day for 14 days.

The magnitude of pain relief could be observed in 2 placebo-controlled studies (10,11). Campbell et al. (10) found that the average reduction in pain intensity was about 58% compared with 28% when they used carbamazepine at a daily dose of 400-800 mg for two weeks. Further, Sturman and O´Brien (11) found a pain reduction of TN in 72% of cases over a period of 24 hours using a maximum daily dose of 3 tablets of 200 mg of carbamazepine (Tegretol®); however, there were side effects in more than half of cases. Similarly, Rockliff and Davis (12) published a placebo-crossover study in which they reported the effectiveness of carbamazepine at doses of 200 mg three times a day to treat TN.

An article by Lechin et al. (13) shows the different results of three active-controlled studies when comparing the use of carbamazepine with pimozide; they found that pimozide reduced TN symptoms in a greater proportion in TN patients, but had more side effects than carbamazepine. On the other hand, the authors of an active-controlled study (14), comparing carbamazepine with tocainide, found that both drugs produced a similar level of relief in TN-associated pain.

Finally, in another active-controlled study in which Vilming et al. (15) compared carbamazepine with tizanidine, the authors found a greater pain relief with carbamazepine in patients with TN; however they observed that tizanidine was better tolerated and had lower side effects than carbamazepine. Nevertheless, they concluded that tizanidine could not be considered effective for the treatment of TN because there was a rapid reemergence of symptoms as soon as the treatment was discontinued.

Finally, 2 systematic reviews on the pharmacological management of TN, published by Wiffen et al. (16) and Jorns et al. (17), concluded that carbamazepine is a drug of first choice because it has proven effective in the treatment of TN. However, both reviews highlighted that there is need for more high-quality studies to draw conclusions based on evidence on the management of TN.

Glossopharyngeal Neuralgia (GN)

The search for “glossopharyngeal neuralgia” and each of the following drugs: “carbamazepine”, “oxcarbamazepine”, “pregabalin”, “gabapentin”, “topiramate”, “etiracetam”, “clonazepam”, “lamotrigine”, “phenytoin”, “vigabatrin”, “phenobarbital” and “valproic acid” in both MEDLINE and COCHRANE databases provided no relevant articles that met the search criteria of this study.

Post-herpetic Neuralgia (PHN)

The search for “post-herpetic neuralgia” and each of the following drugs: “carbamazepine”, “oxcarbamazepine”, “pregabalin”, “gabapentin”, “topiramate”, “etiracetam”, “clonazepam”, “lamotrigine”, “phenytoin”, “vigabatrin”, “phenobarbital” and “valproic acid” in MEDLINE and COCHRANE databases provided 23 articles: 3 articles on “carbamazepine”, 2 articles on “oxcarbamazepine”, 8 articles on “pregabalin”, 6 articles on “gabapentin”, 1 article on “clonazepam”, 2 articles on “lamotrigine”, 1 article on “phenytoin”.

Next, the 23 articles were analyzed to determine if they were pertinent to the issue under study. After this process 14 relevant articles remained under study: 8 randomized clinical trials, 4 systematic reviews, 1 pooled analysis and 1 retrospective study ( Table 4). The 14 articles assessing the effects of different drugs in the treatment of PHN were analyzed according to JADAD and SORT criteria, and only those articles with SORT evidence level 1 or 2, and RCTs with JADAD levels above 3 were included in the study.

**Table 4:**
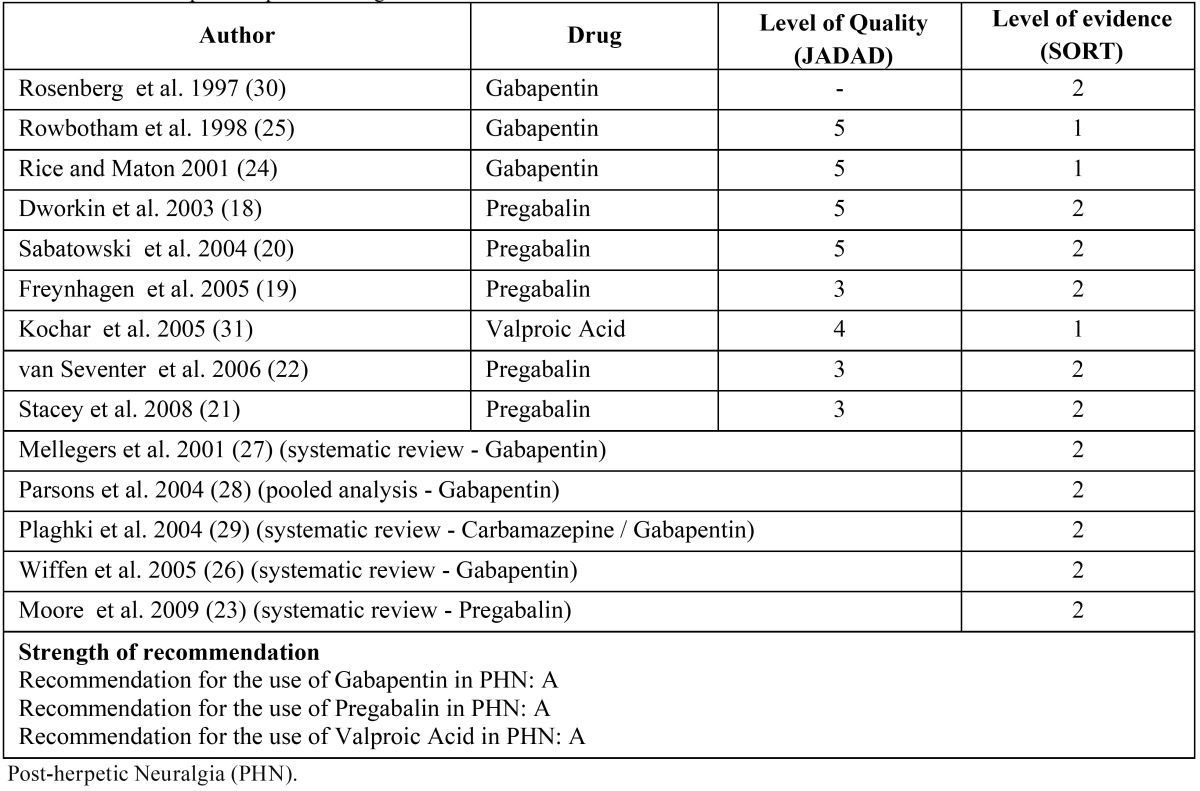
Studies on post-herpetic neuralgia.

Five placebo-controlled RCTs (18-22) assessed the effect of pregabalin to treat the PHN. Doses of 150 mg, 300 mg, 450 mg or 600 mg were found to reduce pain significantly as well as provide a rapid and lasting response. However, in a systematic review published by Moore et al. (23), the authors found that even though pregabalin is not effective at doses of 150 mg, it could be more effective to treat PHN at doses of 600 mg. Nevertheless, the authors pointed out that

the highest benefit is obtained by an individualized treatment aimed at improving the response and minimizing the side effects.

 In one RCT with placebo, Rice and Maton (24) analyzed the effect of gabapentin in patients with PHN and they observed an improvement of pain within one week after starting treatment with a daily dose of 120 mg. They also found fewer cases of sleep disruption when quality of life improved.

On the other hand, a multicenter RCT reported an improvement of symptoms after the second week of treatment to reach a maximum improvement at the fourth week with gabapentin at daily doses of 3600 mg.. No changes in the response were observed at eight weeks of treatment. Side effects included drowsiness, dizziness, ataxia, peripheral edema and infection (25).

A review by Wiffen et al. (26) reported that gabapentin is effective for the treatment of neuropathic pain in patients with PHN. However, the authors claim that there are other drugs, aside from gabapentin, that should be taken into consideration, such as carbamazepine and tricyclic antidepressants to treat PHN.

Similarly, the authors concluded in another review that gabapentin is effective in the treatment of neuropathic pain, but they also added that more studies are necessary to standardize doses and assess possible side effects (27). In a pooled analysis on three RCTs, Parson et al. (28), found that the most frequently side effects of gabapentin were dizziness, drowsiness and peripheral edema, which increased when doses were increased above 180 mg per day. However, dizziness and drowsiness decreased when the dose was increased, and the peripheral edema increased when a maximum dose was administered.

On the other hand, a review published by Plaghki et al. (29) on the pharmacological treatment of PHN reported that the studies included different population groups, and a large variability in both dose and design, a fact that made scientifically impossible to draw conclusions from the data obtained from comparisons between them.

In a retrospective study on the treatment of neuropathic pain with gabapentin in 7 NPH patients, it was observed that an initial daily dose of 1600 mg increased to 2400 per day yielded a 53% of pain reduction. The authors concluded that gabapentin can be used effectively in the treatment of PHN (30).

Finally, in a placebo-controlled RCT on the management of PHN with divalproex sodium, the authors observed a significant recovery in 58% of patients that receive this drug, while this recovery is comparable to that seen with gabapentin, which produced fewer side effects (1 patient). However, the authors concluded that more long term studies with more patients are needed to study the adverse effect of this drug in the long run (31).

Persistent Idiopathic Facial Pain (PIFP)

The search for “persistent idiopathic facial pain”, and “atypical odontalgia”, “phantom tooth pain” and “atypical facial pain”; as well as the drugs “carbamazepine”, “oxcarbamazepine”, “pregabalin”, “gabapentin”, “topiramate”, “etiracetam”, “clonazepam”, “lamotrigine”, “phenytoin”, “vigabatrin”, “phenobarbital”, and “valproic acid” in both the MEDLINE and COCHRANE databases provided 2 articles: one article on “lamotrigine” and another article on “topiramate”. Immediately afterwards, both articles were analyzed to check if they were pertinent to the issue under study. The analysis did not provide any results, which indicated that there is an absence in the literature of good quality articles assessing the pharmacological treatment of PIFP.

Burning Mouth Syndrome (BMS)

The search for “burning mouth syndrome” and each of the following drugs: “carbamazepine”, “oxcarbamazepine”, “pregabalin”, “gabapentin”, “topiramate”, “etiracetam”, “clonazepam”, “lamotrigine”, “phenytoin”, “vigabatrin”, “phenobarbital” and “valproic acid” in both the MEDLINE and COCHRANE databases provided 4 articles in total: 1 article on “carbamazepine”, 3 articles on “clonazepam”. Subsequently, the 4 articles were analyzed to check if they were pertinent to the issue under study. The process brought as a result, 1 relevant article (RCT) ( Table 5).

**Table 5:**
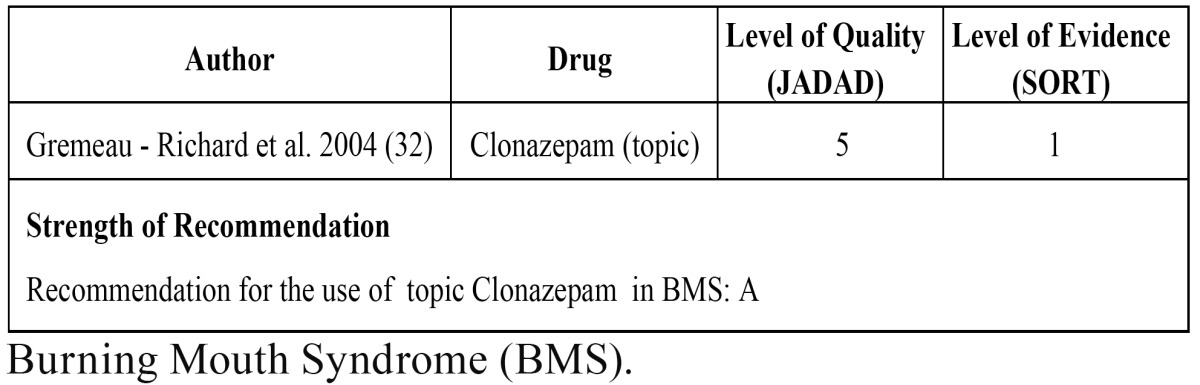
Study for burning mouth syndrome.

The RCT analyzed the effect of topic clonazepam in patients with stomatodynia (BMS), and the authors find that the improvement of pain was significantly higher in the clonazepam group, with a significant decrease of 50% in pain. However, this treatment was not effective in all patients, although the reasons underlying this fact are unknown. The authors concluded that it is essential to deepen our knowledge about the patho-physiological mechanisms of BMS to be able select the best treatment, which includes the topical application of clonazepam and the development of new treatments involving different mechanisms of action. Also, it is necessary to make more high quality studies on the management of this syndrome in order to be able to obtain guidelines on evidence-based treatments, as well as a on good quality patient-oriented evidence (32).

## Discussion

Anticonvulsants were first used for the treatment of NP as determined by the characteristics of temporal profiles and abrupt nature of the painful attacks of this disease, which has similar characteristics to those seen in epileptic seizures (4).

Several studies have supported the use of anticonvulsants for the NP treatment (10-15). In this review we found that carbamazepine is widely used for the TN treatment. We observed that in the study of Lindstrom and Lindblom (14) the dose varies from 100 mg. (10), 200 mg. (12), 400 mg. and 1 g. (8), to a maximum tolerated dose of 20 mg/ kg.

Most of the authors found better results in the treatment of TN with carbamazepine when it was compared with placebo (8-12). However, in 3 studies comparing the effect of this medication with other drugs they obtained different results. Lindstrom and Lindblom (14) compared carbamazepine with tocainide and obtained similar results in pain relief; Lechin et al. (13), obtained a greater pain relief with pimozide than with carbamazepine; Vilming et al. (15), compared carbamazepine with tizanidine and they found that carbamazepine was more effective. Nowadays there are no guidelines about a standard dose for the treatment of TN, suggesting that the dose would be related to the patient’s needs and response.

However, as we have seen in this review on the NT treatment, carbamazepine is a drug of first choice. The control and monitoring of these patients is of the utmost interest (4). Oxcarbazepine should be administered as a second-choice drug in those cases when there is a poor control of the pain, or adverse effects occur (10). Because studies included in this review on the TN treatment show consistent evidence, the strength of recommendation for the use of carbamazepine in the TN is type A.

As we observed, there are no high quality studies evaluating the effect of newer drugs for the treatment of NP and especially the TN. For the PHN treatment with gabapentin, Rosenberg et al. (30), administered a maximum dose of 2400 mg per day and observed a pain reduction close to 53% of patients with PHN, so they concluded that gabapentin could be used effectively.

Rice et al. (24) carried out a clinical trial on the PHN treatment with gabapentin at different doses. They found more effective results in pain reduction with doses of 1200 mg; therefore, they concluded that gabapentin is effective at doses of 1200 mg per day in the control of PHN-induced pain, and also observed an improvement of sleep quality and quality of life in these patients.

In another study, the authors observed progress of PHN patients treated with gabapentin at doses of 3600 mg per day, as well as a good response in 43% of the 229 patients enrolled in the study (25).

In a review on the pharmacologic management of patients with PHN, the authors reported that gabapentin was effective at daily doses of 1800 mg and 2400 mg. However, the authors report side effects, such as dizziness and drowsiness. So they concluded that gabapentin is both safe and effective and could be used routinely (29).

However, in a pooled analysis of adverse effects detected in three clinical trials, which included patients with post-herpetic neuralgia, Parson et al. (28) observed that the incidence of peripheral edema increased when the dose exceeded 1800 mg per day and also that the commonest side effects were, in a transient manner, dizziness and drowsiness. They concluded that gabapentin is safe and does not have any inconveniences that may limit this dose to achieve an optimum efficacy (28).

Finally, in a review by Mellegers et al. (27), the authors conclude that this drug is effective, but its efficacy may be reduced if it is administered at very low doses. On the other hand, they point out that when doses are rapidly increased, CNS adverse effects do also increase.

As we observed in this review, gabapentin has been rated by several quality studies as the drug of choice for the treatment of PHN patients. All studies reported significant pain relief, improved quality of life and sleep, which therefore shows that it is an effective drug. However, it should be administered gradually to reduce side effects (24).

So, bearing in mind the consistent evidence found in the studies, the strength of recommendation that we suggest for the use of gabapentin in the treatment of PHN is type A.

It was observed that pregabalin produced a significant pain relief in the treatment of PHN patients. Doses varied and pain relief was significant in all cases (18–22). Nevertheless, Moore et al. (23) found in their systematic review that daily doses at 150 mg are not effective, although 600 mg doses per day are indeed effective for pain relief, but present more side effects (dizziness and drowsiness). The authors recommend a flexible-dosing regimen aimed at adjusting drug doses and optimizing their safety and efficacy (19). So, the strength of recommendation for the use of pregabalin in the treatment of PHN patients is, on account of the consistent evidence shown in the studies, type A.

There are few studies documenting the effectiveness of valproic acid (VA) in the treatment of neuropathic pain (4). In this review we only found one high quality RCT assessing the effect of VA in PHN. Kochar et al. (31) observed a significant pain reduction in most patients at doses of 1000 mg of divalproex sodium per day for 8 weeks, so they concluded that valproic acid could be comparable to gabapentin in its effectiveness to achieve pain relief; they also manifest that this drug has a good tolerance and could used as an alternative drug to gabapentin. However, they also added that further studies with more patients and longer duration will be needed to assess the effects of valproic acid in the long run.

The strength recommendation for the use of valproic acid in the treatment of PHN is, on account of the consistent evidence shown by the studies show, type A.

 As to BMS, we only found one high-quality article that assessed topical application of clonazepam (chewable tablets of 1 mg of clonazepam held in the mouth for 3 minutes). The authors found pain relief to be about of 50% when compared with placebo; however, they found that not all patients responded with the same the same level of efficiency, a fact that would result in the need for more quality studies aimed at evaluating the effects of topical clonazepam to treat BMS (32).

The strength recommendation for the use of topical clonazepam in the BMS is, on the basis of this high-quality study, type A.

As for other NPs, such as GN and PIFP, we did not find any high-quality studies on the use of any specific drug to treat this disorders.

In conclusion, carbamazepine is the first drug of choice for the treatment of TN. Our recommendation is, with regard to the consistent evidence found in the studies, type A. As to the treatment of PHN, we have a good number of high-quality studies that assess the effects of three drugs (gabapentin, pregabalin and valproic acid); all of them have been found to be effective in the relief of symptoms, so the strength recommendation for pregabalin, pregabalin and valproic acid is, as determined by the aforementioned high-quality studies, type A. As for the treatment of BMS, we found one high-quality study that demonstrates the effectiveness of topical clonazepam in the treatment of this disease, so our recommendation for the use of this drug in BMS is, on the basis of abovementioned study, type A.

Our recommendations are based on quality studies; however, the design of them is variable, so it would be necessary a standardization of designs to carry out valuable comparative analyses in future studies.

We also observed that there is a need for higher-quality studies that may assess the effectiveness of the different anticonvulsants used in the treatment of NP, and thus be able to make a comparative study between the different drugs.
